# Vitamin E protects from lipid peroxidation during winter stress in the seagrass *Cymodocea nodosa*

**DOI:** 10.1007/s00425-022-03825-2

**Published:** 2022-01-17

**Authors:** Sergi Munné-Bosch, Sandra Puig, Erola Fenollosa, Andrea Casadesús, Estrella Fernández

**Affiliations:** 1 Department of Evolutionary Biology, Ecology and Environmental Sciences, University of Barcelona, Av. Diagonal 643, 08028 Barcelona, Spain; 2 Research Institute of Biodiversity (IrBio), Faculty of Biology, University of Barcelona, Av. Diagonal 643, 08028 Barcelona, Spain

**Keywords:** Antioxidants, Cold stress, Lipid hydroperoxides, Low temperatures, Malondialdehyde, Marine angiosperms, Tocopherols

## Abstract

**Main conclusion:**

Adjustments in the antenna size and α-tocopherol contents provide protection from sustained damage in leaves of a seagrass, while low vitamin E contents appear to be enough to protect rhizomes (which appear to be more cold tolerant than leaves).

**Abstract:**

Despite low temperatures can adversely affect the proper growth and development of marine angiosperms, by, among other processes, increasing reactive oxygen species production and causing oxidative damage to lipid membranes, the role of vitamin E in seagrasses, such as *Cymodocea nodosa* has not been explored thus far. Here, we aimed to better understand the possible role of this chain-breaking (peroxyl radical-trapping) antioxidant in response to low temperatures, and most particularly in relation to the occurrence of photo-inhibition and lipid peroxidation. Low temperatures caused an important desiccation of leaves, but not of rhizomes, which were much more tolerant to cold stress than leaves. Cold stress during winter was associated with chlorophyll loss and transient photo-inhibition, as indicated by reversible reductions in the *F*_v_/*F*_m_ ratio. Adjustments in pigment antenna size and vitamin E contents per unit of chlorophyll during winter may help protect the photosynthetic apparatus from sustained photo-inhibitory damage and lipid peroxidation events in leaves. Rhizomes also accumulated significant amounts of vitamin E, although to a much lesser extent than leaves, and kept protected from lipid peroxidation during winter, as indicated by malondialdehyde contents, a product from secondary lipid peroxidation. It is concluded that vitamin E can help protect both leaves and rhizomes from lipid peroxidation, although cold stress during winter can cause transient photo-inhibition of the photosynthetic apparatus, in *C. nodosa*.

## Introduction

Marine prairies are widely distributed in all but coastal areas of the world, except around the polar regions. They occupy an area of 3.45 × 10^5^ km^2^ which represents up to 0.1–0.2% of total coastal oceans (Banerjee et al. 2018), being one of the most productive ecosystems which are found in coastal areas (Bracun 2016). In addition, they play an important role in the trophic network and provide many goods and services to society (Barbier et al. 2011). Furthermore, marine prairies are bio-constructive ecological ecosystems providing habitat to many species for feeding, reproduction and protection and therefore incorporate a great biological diversity (Hemminga and Duarte 2000). On the other hand, they participate in the carbon cycle by capturing a high amount of carbon dioxide, act as sediment stabilizers and improve sedimentation or particulate matter (Bracun 2016). The lattice structure of rhizomes from these plants allows stabilizing the substrate, helps reduce the effects of erosion of coastline and regression of beaches, and reduces the hydrodynamics of currents and waves (Van der Heide et al. 2012; Pérez et al. 2015).

In the last 50 years, due to the increase in anthropogenic pressures on these ecosystems, an increase in losses of marine prairies has been observed in both tropical and temperate regions (Orth et al. 2006). It has been estimated that the rate of decline of marine prairies on a global scale reached 1% per year before 1940 and 5% per year after 1980 (Waycott et al. 2009). Acceleration of its degradation and decrease on a global scale results from a combination of different pressures arising from human activity: climate change (e.g., increase in temperature, sea level rise, increase in frequency and intensity of drastic climatic events), alteration of water quality, increase in nutrients, sediments and pollutants, destruction of plants by commercial fishing practices and boat anchorages, destruction of habitat for construction of human infrastructure, regeneration of beaches or introduction of non-native species, among others (Boudouresque et al. 2009; Romero et al. 2015).

Plants growing under optimal conditions are constantly producing reactive oxygen species (ROS) as part of the correct functioning of their aerobic metabolism (Hasanuzzaman et al. 2020), and the presence of ROS at low concentrations, it is important because they are involved in signaling networks deployed to activate various physiological processes (Xie et al. 2019). ROS are one of the most important components of the signaling cascade activated by plants in response to cold, and its sustained production is essential for the correct regulation of gene expression under stressful conditions (Yadav 2010). However, when produced at high concentrations in a sustained manner, ROS can cause peroxidation of lipid membranes, protein oxidation, enzyme inactivation, nucleic acid damage and even lead to cell death (Krieger-Liszkay and Trebst 2006). Thylakoids are very prone to lipid peroxidation since chloroplasts function at high oxygen tensions and in the light, so that photooxidative processes due an absorption of excess of energy can easily occur (Asada 2006). Although they are also generated in other compartments as in mitochondria, peroxisomes, plasma membrane and cell wall (Hasanuzzaman et al. 2020), ROS produced in chloroplasts can be one of the main causes of cell death if not eliminated by antioxidants.

One of the most important chloroplastic antioxidants is α-tocopherol (α-toc), which belongs to the vitamin E group of compounds. α-Tocopherol, which is the major vitamin E form present in photosynthetic tissues, is made of an aromatic ring with a hydroxyl group (chromanol), which readily reacts with lipid peroxyl radicals and ROS (mainly singlet oxygen), and a long hydrophobic chain (polyprenoid chain), which anchor the molecule to the thylakoid membrane (Krieger-Liszkay and Trebst 2006; Falk and Munné-Bosch 2010; Muñoz and Munné-Bosch 2019; Hasanuzzaman et al. 2020). α-Tocopherol efficiently eliminates singlet oxygen both by (physical) quenching and (chemical) scavenging, and it is the only antioxidant present in plant cells capable of inhibiting the propagation of lipid peroxidation, so its function within chloroplasts is essential to protect thylakoids from lipid peroxidation (Muñoz and Munné-Bosch 2019). β-Carotene can help α-toc in protecting the photosynthetic apparatus from singlet oxygen (Trebst 2003) and ascorbate is essential to recycle α-tocopheroxyl radicals, which are the products of the action of α-toc scavenging lipid peroxyl radicals (Munné-Bosch and Alegre 2002; Kanwischer et al. 2005). The function of α-toc as an inhibitor of photo-inhibition and in maintaining the stability of the lipid membranes has been studied in cyanobacteria and terrestrial plants (Havaux et al. 2005; Inoue et al. 2011; Kumar et al. 2021), but to our knowledge, no studies have been performed thus far on its role in marine angiosperms.

Marine angiosperms are grouped into four different families (Posidoniaceae, Cymodoceaceae, Zosteraceae and Hydrocharitaceae) that converged with the development of different adaptations for marine life. Nowadays, *Cymodocea nodosa* is one of the most representative marine plant species in the Mediterranean Sea that belongs to this group of higher plants of terrestrial origin that colonized the middle sea about 100 million years ago (Pérez et al. 2015). Here, we aimed at better understanding mechanisms underlying cold stress tolerance in *C. nodosa*, with a focus on studying the involvement of vitamin E in the protection from lipid peroxidation both in photosynthetic tissues (leaves) and non-photosynthetic tissues (rhizomes). We hypothesized that vitamin E might accumulate in leaves, but not in rhizomes, in response to winter stress to specifically protect the photosynthetic apparatus from cold-induced photo-oxidative stress.

## Materials and methods

### Plant material and sampling

The study was carried out in the Alfacs Bay at the Ebro delta in the north-eastern coast of Spain, in the province of Tarragona, Catalonia (NE Spain, 40° 35′ 48.8″ N 0° 42′ 52.2″ E). Alfacs Bay, where *C. nodosa* is the dominant marine angiosperm species, is an estuarine embayment in the coast that is shallow (maximum depth 6 m) and is closed to the Mediterranean Sea by a long, narrow sand bar dominated by nutrient inputs from channels collecting the runoff from neighboring rice paddy fields (Pérez et al. 1994). Samples were collected from *C. nodosa* plants found in the shallowest areas (more specifically at 2 m depth) during February 25th (winter), June 3rd (spring) and August 1st (summer) 2019. All samplings were always performed on fully expanded young leaves and rhizomes of 15 plants (at least 2 m apart) on sunny days at midday. In addition, every day of sampling, measurements of the environmental conditions of the water (temperature, pH, and conductivity) were taken. For each individual five leaves and five pieces of rhizome were taken, avoiding the ends and extracting all epiphytes before measurements. All samples were frozen with liquid nitrogen and stored at − 80 °C until analyses. In addition, a leaf and a piece of rhizome were additionally separated from each individual in a falcon tube with water from the study area and kept in the dark to estimate the leaf hydration and *F*_v_*/F*_m_ ratio upon arrival to the laboratory.

### Leaf hydration, chlorophyll contents and *F*_v_/*F*_m_ ratio

Upon arrival to the laboratory (around 3 h from samplings), samples were weighed with a precision balance to obtain the fresh mass. Then, chlorophyll fluorescence was measured in leaves (which previously adapted to darkness for 3 h) using a mini-PAM (Walz, Effeltrich, Germany) and the maximum efficiency of PSII photochemistry (*F*_v_/*F*_m_ ratio) estimated following van Kooten and Snel (1990). Both foliar and rhizome samples were then dried at 70 °C to constant weight to measure the dry mass. Leaf hydration (*H*) was then calculated as (fresh mass − dry mass)/dry mass. For pigment estimation, frozen samples were grounded and repeatedly extracted with methanol containing 0.01% butylated hydroxytoluene (BHT; w/v), using vortex for 20 s followed by 30 min of ultra-sonication (Bransonic ultrasonic bath 2800, Emerson Industrial, Danbury, CT, USA) during each extraction, until the final pellet was colorless. Then, supernatants were pooled and centrifuged at 1419*g* during 10 min at 4 °C and chlorophyll (Chl) *a* + *b *content and the Chl *a*/*b* ratio were determined spectrophotometrically using the equations described by Lichtenthaler (1987).

### Lipid peroxidation analyses

Lipid peroxidation levels were determined by analyzing primary (lipid hydroperoxide) and secondary (malondialdehyde, MDA) lipid peroxidation products. First, for evaluating lipid hydroperoxides content, frozen samples were extracted with methanol containing 0.01% BHT (w/v) at 4 °C using 30 min of ultra-sonication (Bransonic ultrasonic bath 2800) and centrifugation at 1419*g* for 10 min at 4 °C. Then, two re-extractions were performed. Supernatants were pooled and used for analyses using the Fox-2 reagent (consisting in a solution of 90% methanol (v/v) containing 25 mM sulfuric acid, 4 mM BHT, 0.25 mM iron sulfate ammonium (II) and 0.1 mM xylenol orange) as described in Bou et al. (2008). Absorbances were measured at 560 and 800 nm. A calibration curve using hydrogen peroxide 37% (v/v) was used for quantification.

For determining the MDA content, the thiobarbituric acid-reactive substances assay, was used as in Hodges et al. (1999). In brief, samples were extracted with 80% (v/v) ethanol containing 0.01% (w/v) BHT, then vortexed for 20 s and exposed to ultrasonication for 30 min (Bransonic ultrasonic bath 2800). After a centrifugation process at room temperature for 13 min at 1091*g*, the supernatant was recovered, and the pellet re-extracted twice using the same procedure. Then, two tubes for sample were used: (i) − thiobarbituric acid (TBA), with 1 mL extract + 1 mL 20% trichloroacetic acid (w/v) with 0.01% BHT (w/v) and (ii) + TBA, with 1 mL extract + 1 mL 20% trichloroacetic acid (w/v), 0.01% BHT (w/v) and 0.65% TBA (w/v). Tubes were incubated at 95 °C for 25 min. Subsequently, the reaction was stopped by maintaining them at 4 °C for 10 min. After centrifugation at 1091*g* at room temperature for 5 min, MDA content in samples were analyzed by spectrophotometry and quantified using the equations developed by Hodges et al. (1999).

### Vitamin E analyses

The quantification of the different homologues of tocopherols and tocotrienols (α, β, γ and δ) was performed as follows. Samples were extracted with methanol containing 0.01% BHT (w/v). Samples were extracted and re-extracted twice using vortex for 20 s followed by 30 min of ultrasonication (Bransonic ultrasonic bath 2800). Then, supernatants were pooled and centrifuged at 1419*g* during 10 min at 4 °C before passing them onto a hydrophobic PTFE filter 0.22 μm (Phenomenex, Torrance, CA, USA) and introducing them into vials. Vitamin E compounds were separated by HPLC at room temperature using an Inertsil 100A column (5 μm, 0.03 × 0.25 m, GL Sciences Inc., Tokyo, Japan) and quantified with a fluorescent detector, as described by Amaral et al. (2005). A calibration curve was made with authentic standards (Sigma-Aldrich, Steinheim, Germany) for each of the tocopherols and tocotrienols analyzed.

### Statistical analyses

Statistical analyses were performed by applying a one-way ANOVA and indicating Duncan post hoc differences with different letters among different times of measurements (IBS SPSS Statistics 19; SPSS Inc., IL, USA). Differences were considered significant when *P* values were below 0.05 (*P* < 0.05).

## Results

### Cold stress causes desiccation during winter stress in leaves, but not in rhizomes

**Fig. 1:**
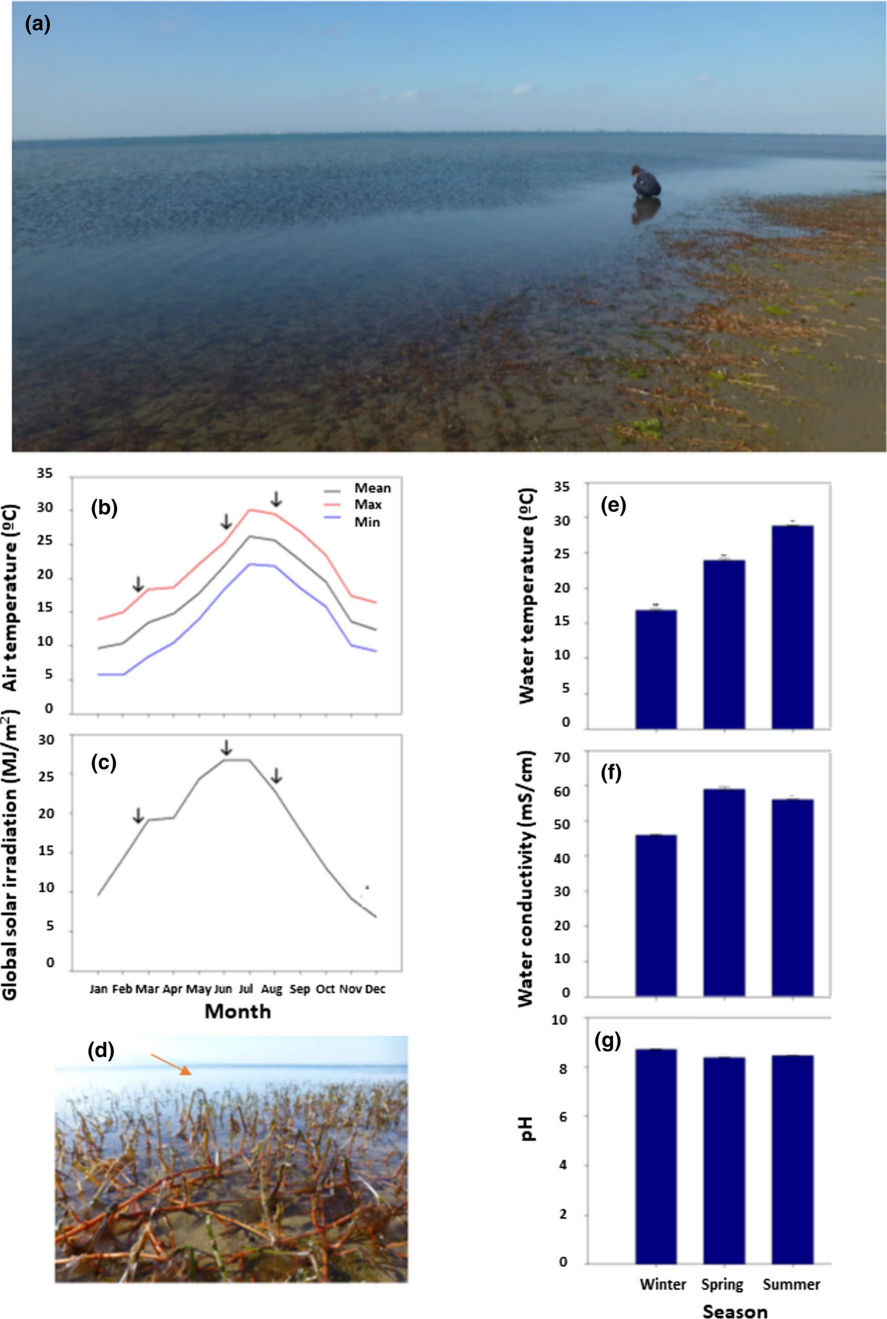
Environmental and site characteristics of the natural ecosystem of the Alfacs Bay where *C. nodosa* was studied. **a** Photograph of the study site. **b**, **c** Mean monthly air temperature and global solar radiation in the Alfacs Bay during the year of the study. **d** Experimental site where *C. nodosa* plants were collected for the study. **e**–**g** Water temperature, conductivity and pH during the samplings performed in winter, spring and summer. Data are the mean SE of *n* = 5

**Fig. 2:**
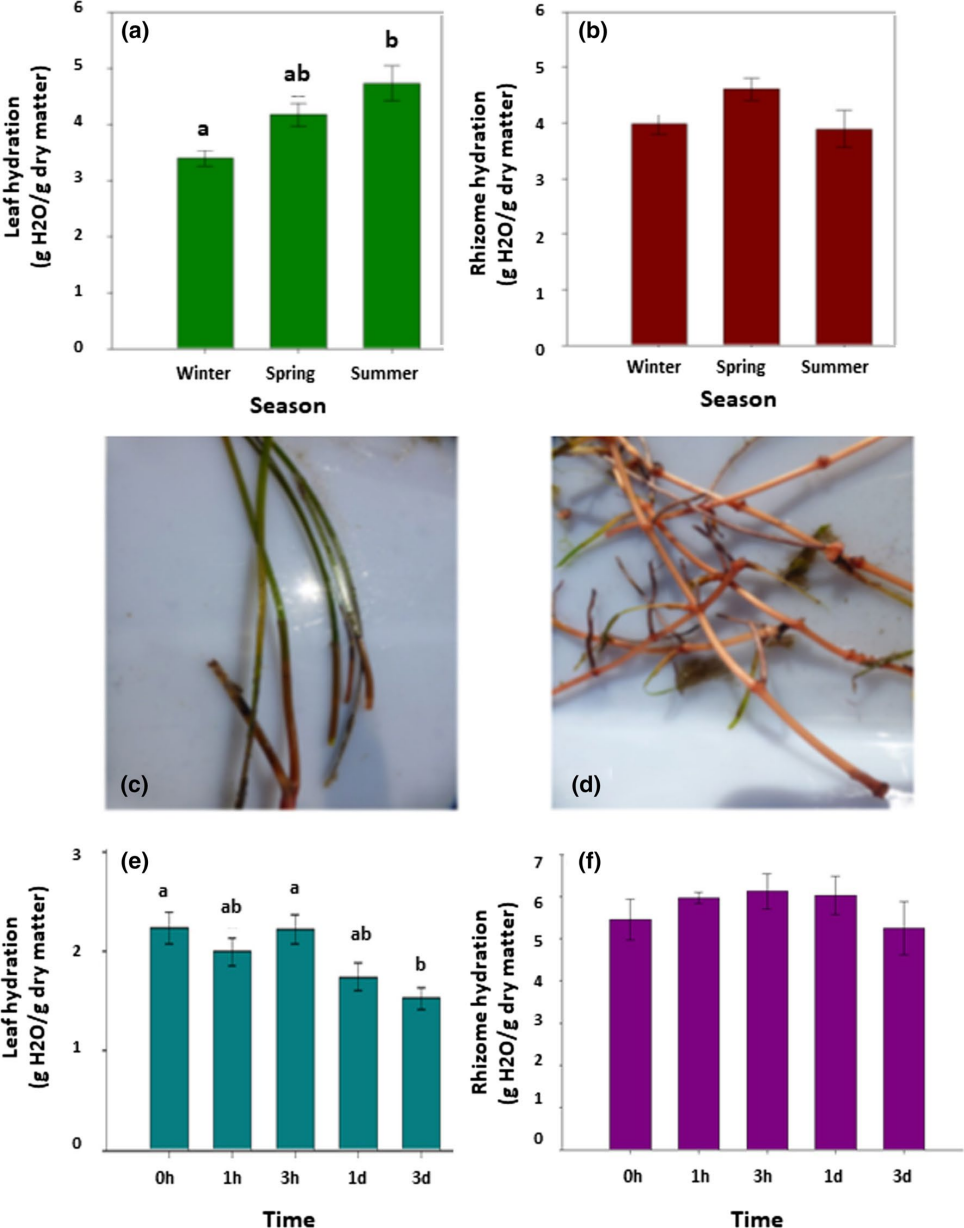
Water content of *C. nodosa* plants. **a**, **b** Seasonal variations in leaf and rhizome hydration in Alfacs Bay. **c**, **d** Detail of leaves and rhizomes used in the study of water loss under controlled conditions. **e**, **f** Dynamics of leaf and rhizome hydration during exposure to 8 °C in a cold chamber. Data are the mean SE of *n* = 15 for the natural site and *n* = 5 for controlled conditions. Different letters indicate statistical differences between sampling time points (Duncan post hoc tests, one-way ANOVA, *P* < 0.05)

*Cymodocea nodosa* plants collected from shallow areas at 2 m depth in the Ebro delta (Fig. 1a) were subjected to contrasting environmental conditions during the year. This area was characterized by minimum and maximum monthly air temperatures of 5 °C and 30 °C during winter and summer, respectively (Fig. 1b), periods that coincided with relatively low and very high global solar irradiation received during the year (Fig. 1c). During the sampling of winter (February 25, 2019), which was performed at midday, plants were under water at 2 m depth (at about 100 m from the coastline, Fig. 1d) and exposed to a water temperature of 15.3 °C (Fig. 1e), conductivity of 50 mS/cm (Fig. 1f) and pH of 8.5 (Fig. 1g). During the next samplings of spring and summer, air and water temperature increased progressively (Fig. 1b, e), global solar irradiation attained its maximum yearly values (Fig. 1c) and water conductivity was 20–30% higher (Fig. 1f), whereas pH was only very slightly lower, with values always above 8 (Fig. 1e). In other words, plants were subject to changing physical variables, but water temperature was most altered during the two sampling extremes since it doubled from winter to summer (Fig. 1e). Tissue hydration measurements revealed that cold stress during winter caused leaf but not rhizome desiccation. Hydration, with values of 3.2 and 4 g H_2_O/g dry matter in leaves and rhizomes, respectively, recovered during spring and summer in leaves, but remained constant in rhizomes during the same period (Fig. 2a, b). Cold-induced desiccation was confirmed by exposing winter leaves (Fig. 2c) and rhizomes (Fig. 2d) to a water temperature of 8 °C under laboratory conditions (by exposing plants to cold stress in a cold chamber for 3 days in seawater). In this experiment, it was observed that hydration decreased progressively during cold stress in leaves (particularly after one day of cold stress exposure, Fig. 2e), while rhizomes kept their water contents (Fig. 2f). To sum up, leaves of *C. nodosa* plants in the Ebro delta were exposed to cold stress during winter, an abiotic factor that induced leaf desiccation as well, while the same environmental conditions did not induce desiccation of rhizomes (the perennial organ).

### Chlorophyll loss, photo-inhibition and vitamin E during winter stress

**Fig. 3:**
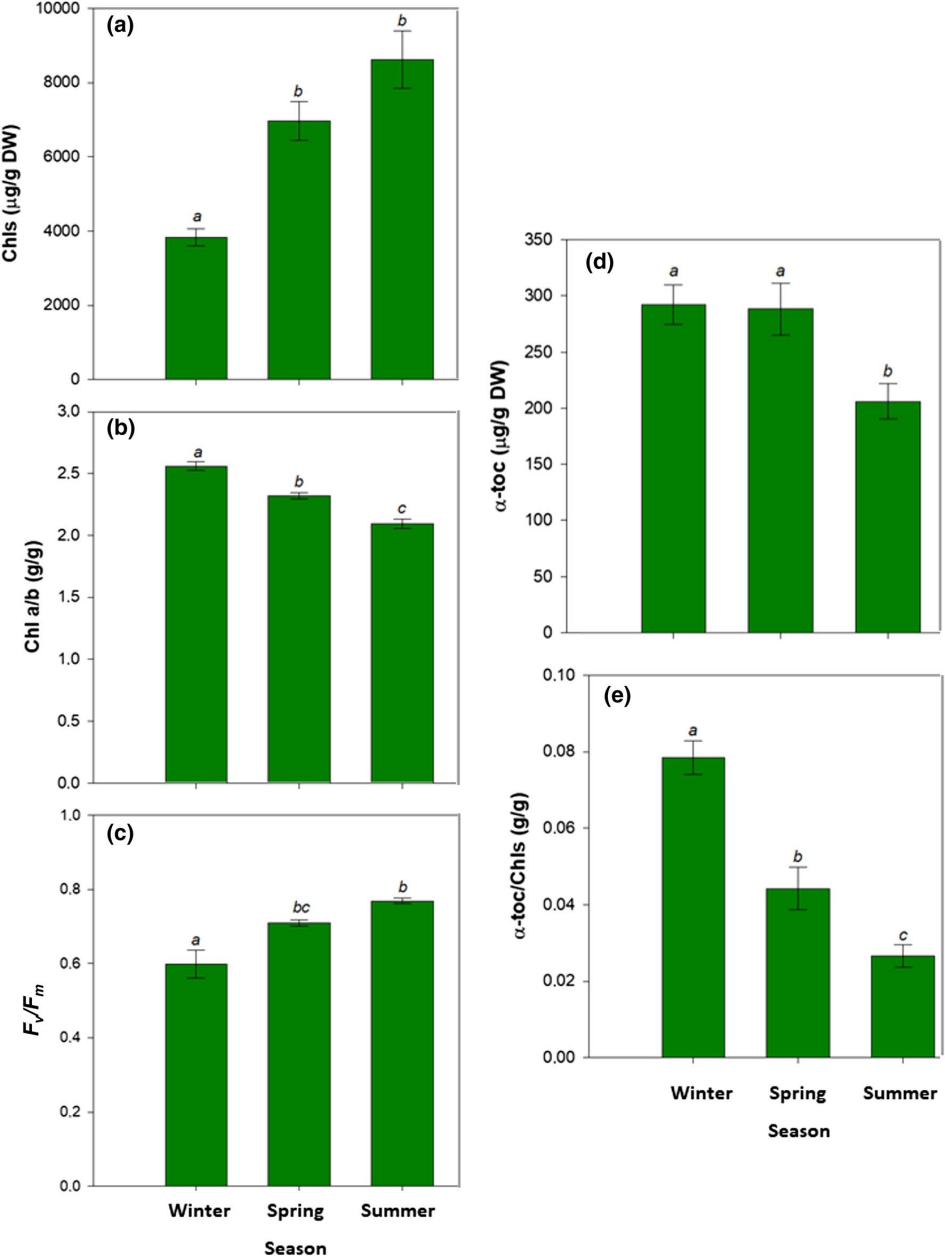
Seasonal variations in chlorophyll contents, photoinhibition and vitamin E contents in leaves of *C. nodosa*. **a** Chlorophyll *a* + *b* (Chls) content, **b** Chl *a*/*b* ratio, **c** maximum efficiency of PSII photochemistry, **d** α-tocopherol (α-toc) content and **e** α-toc/Chls ratio of leaves of *C. nodosa* plants in the Alfacs Bay during winter, spring and summer. Data are the mean SE of *n* = 15. Different letters indicate statistical differences between sampling time points (Duncan post hoc tests, one-way ANOVA, *P* < 0.05)

Chls contents in leaves during winter averaged 3.95 mg/g dry matter, and they increased progressively during spring and summer to attain values up to 8.50 mg/g dry matter, that is more than twofold higher in summer compared to winter (Fig. 3a). Seasonal variations also revealed higher Chl *a*/*b* and lower *F*_v_/*F*_m_ values during winter compared to summer, with Chl *a*/*b* values decreasing from 2.5 to 2.1 g/g and *F*_v_/*F*_m_ ratios increasing from 0.59 to 0.78 from winter to summer, respectively (Fig. 3b, c). It noteworthy that *F*_v_/*F*_m_ values averaged values below 0.75, which is the threshold indicating photoinhibition (see Takahashi and Badger 2011), both during winter and spring (Fig. 3c), paralleling variations in leaf hydration (Fig. 2a) and water temperature (Fig. 1e).

**Fig. 4:**
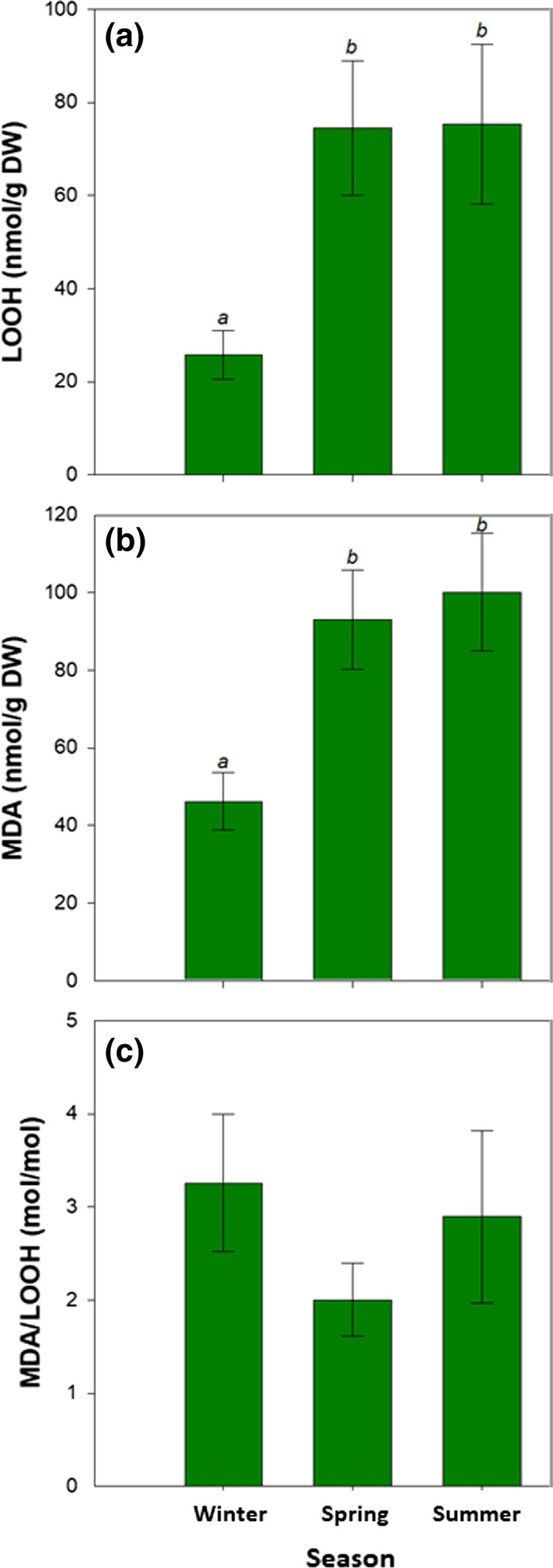
Seasonal variations in the extent of lipid peroxidation in leaves of *C. nodosa*. **a** Contents of lipid hydroperoxides (LOOH), a primary lipid peroxidation product, **b** malondialdehyde (MDA), a secondary product of lipid peroxidation, and **c** LOOH/MDA ratio in leaves of *C. nodosa* plants in the Alfacs Bay during winter, spring and summer. Data are the mean SE of *n* = 15. Different letters indicate statistical differences between sampling time points (Duncan post hoc tests, one-way ANOVA, *P* < 0.05)

Vitamin E analyses revealed that *C. nodosa* leaves contained tocopherols, but not tocotrienols, and the major tocopherol was in the form of α-tocopherol (α-toc), which was found at a concentration of 290 μg/g dry matter in winter (Fig. 3d). γ-Tocopherol was found in some samples only, and when present it was always present at very low, not quantifiable amounts (traces). β- and δ-tocopherols and all tocotrienols were not detected in any sample (detection limit < 1 μg/g dry matter). Therefore, α-toc accounted always above 99% of the total vitamin E content. While *F*_v_/*F*_m_ ratio increased progressively between winter and summer (Fig. 2c), α-toc content followed a different trend keeping higher both during winter and spring compared to summer (Fig. 3d). In contrast, the α-toc content expressed on a Chl basis did vary similarly to the Chl *a*/*b* ratio (Fig. 3b) and inversely to the *F*_v_/*F*_m_ ratio (Fig. 3c), with the maximum α-toc/Chl ratios attained during winter (0.079 g/g Chl, which is equivalent to 79 mg/g Chl, Fig. 3e). Noteworthy, the maximum α-toc/Chl ratios coincided with the lowest contents of primary (LOOH) and secondary (MDA) lipid peroxidation products during winter (Fig. 4a, b). Despite the LOOH/MDA ratio kept constant during the study (Fig. 4c), LOOH and MDA contents were much lower during winter than during spring and summer (Fig. 4a, b).

### Vitamin E and lipid peroxidation in rhizomes

**Fig. 5:**
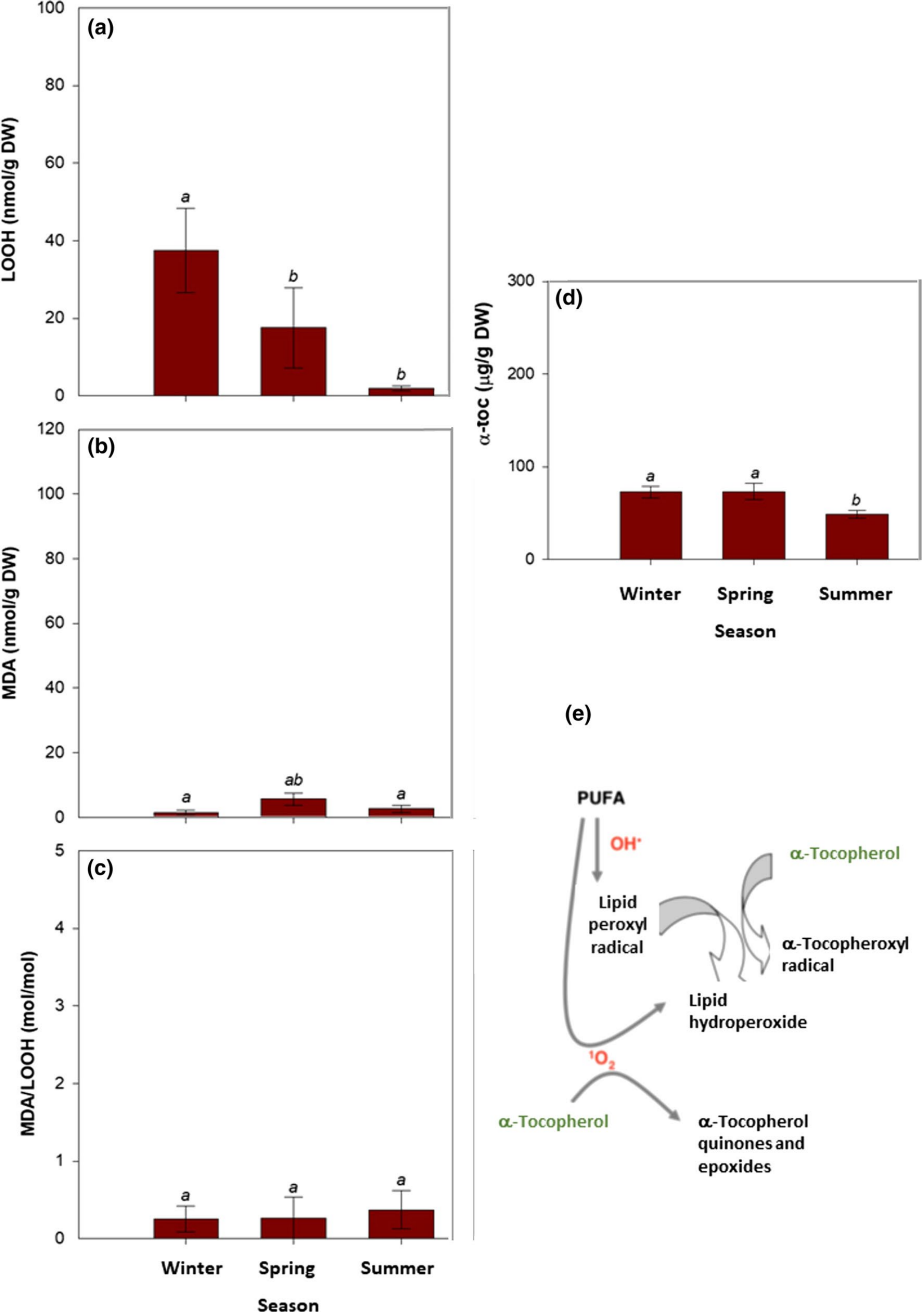
Seasonal variations in the extent of lipid peroxidation and vitamin E content in rhizomes of *C. nodosa* in the Alfacs Bay during winter, spring and summer. **a** Contents of lipid hydroperoxides (LOOH), a primary lipid peroxidation product, **b** malondialdehyde (MDA), a secondary product of lipid peroxidation, **c** LOOH/MDA ratio, and **d** α-tocopherol (α-toc). **e** Antioxidant function of α-toc as a chain-breaking antioxidant reacting with lipid peroxyl radicals and hence inhibiting the propagation of lipid peroxidation. Note that this reaction gives rise to the production of LOOH. Data are the mean SE of *n* = 15. Different letters indicate statistical differences between sampling time points (Duncan post hoc tests, one-way ANOVA, *P* < 0.05)

The extent of lipid peroxidation in rhizomes was in general lower compared to leaves, at least in terms of LOOH and MDA accumulation, and it also followed a completely different trend. LOOH content in rhizomes peaked at winter, with values reaching 38 nmol/g dry matter (Fig. 5a). MDA content in rhizomes was constant over the seasons and kept very low, always with mean values below 5 nmol/g dry matter (Fig. 5b). The MDA/LOOH ratio in rhizomes also kept at constant levels between samplings (Fig. 5c), as it occurred in leaves (Fig. 4c), but always at levels 90% lower than in leaves. Vitamin E contents in rhizomes, also represented by more than 99% of α-toc, were also much lower than in leaves, with α-toc contents being slightly higher in winter and spring, compared to summer (Fig. 5d). It is noteworthy that the scavenging activity of α-toc against lipid peroxyl radicals to prevent the propagation of lipid peroxidation in thylakoid membranes prevents MDA production, but this activity itself leads to LOOH production (Fig. 5e, see also Muñoz and Munné-Bosch 2019).

## Discussion

The five species of marine angiosperms that inhabit the Western Mediterranean are *Posidonia oceanica*, *Cymodocea nodosa*, *Zostera marina*, *Zostera noltii* and *Ruppia cirrhosa* (Romero et al. 2015). Most of the populations of *C. nodosa* are distributed from along the Spanish coasts of the Mediterranean, but they can also be found along the Atlantic coasts of southern Portugal and the northeast Africa (Pérez et al. 2015). Being the second marine angiosperm of greater extension of the Spanish coasts after *P. oceanica* (Ruiz et al. 2015), *C. nodosa* is a species of warm water that lives in sand or mud bottoms of shallow areas (Boudouresque et al. 2009), although it can also be present in less dense populations in deeper waters (Romero et al. 2015). Depending on the geographical area, it can reach a maximum depth that varies between 15 and 36 m (Pérez et al. 2015). The populations that are in shallow areas, due to the high intensity of the sea current, are usually more discontinuous populations, unlike what is observed in the populations of greater depth, and phenotypic plasticity provides them tolerance to a wide range of environmental conditions (Pérez et al. 2015). This allows *C. nodosa* to grow in areas where temperature and salinity conditions can reach extreme values (Pérez et al. 2015). For example, populations have been mapped in Alfacs Bay, where minimum and maximum temperatures can reach 7 °C and 30 °C, respectively (Romero et al. 2015). In addition, it has been shown that this species can tolerate a wide range of salinity levels (Pérez et al. 2015), as well as seasonal variations that cause changes in light, temperature, and conditions of nutrient discharge (Marbà et al. 1996; Guidetti et al. 2002; Bracun 2016). *C. nodosa* has a great ability to colonize new areas in a short time due to the huge capacity of the rhizome for rapid growth, except in the winter months when this growth capacity is reduced (Duarte et al. 2006). Here, it is shown that the rhizome has a greater capacity than leaves to tolerate low-temperature stress, being the rhizome more desiccation tolerant than leaves and showing a lower extent of lipid peroxidation, as indicated by lower MDA contents.

**Fig. 6:**
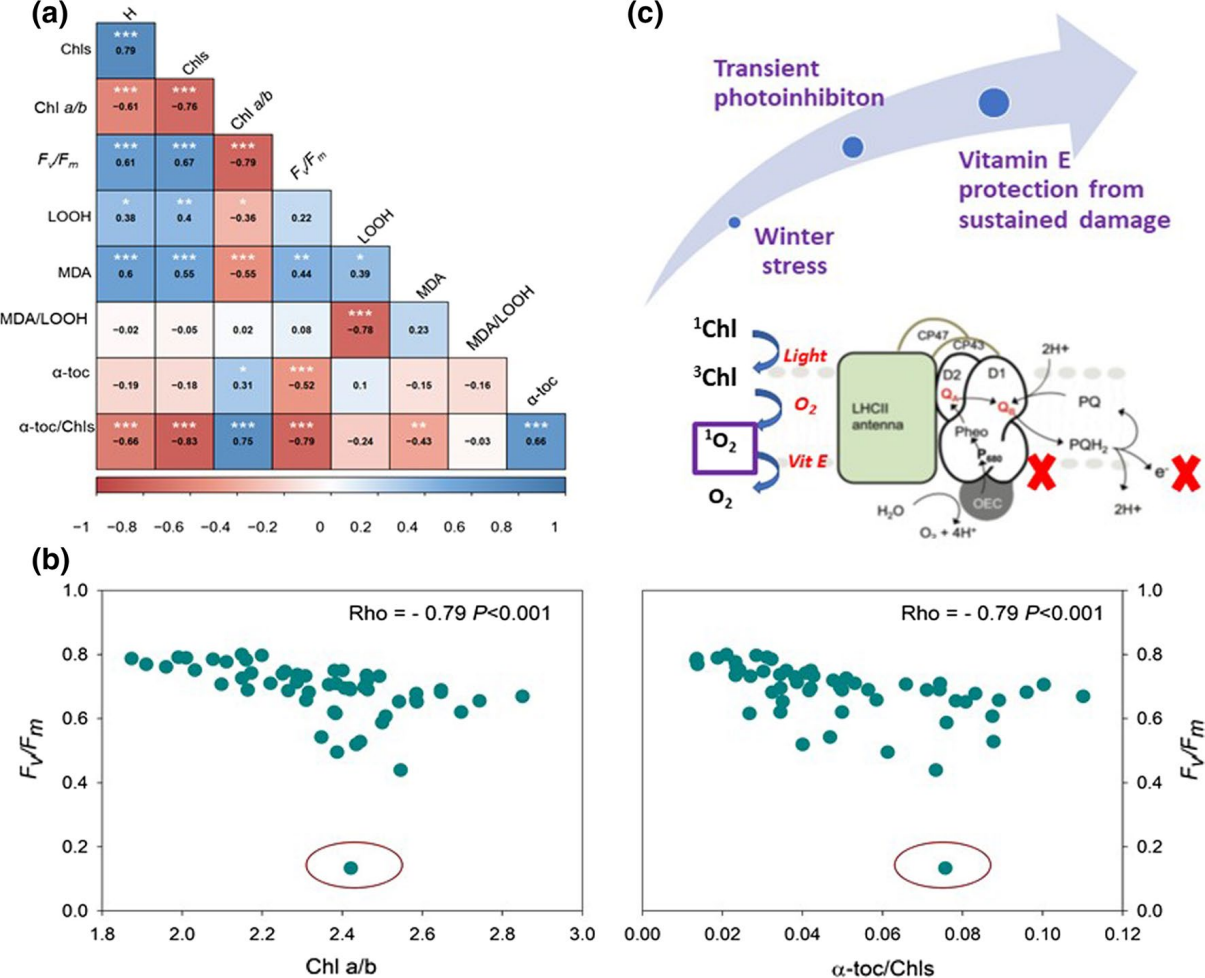
Correlations and model depicting of action of vitamin E in the protection of *C. nodosa* leaves from winter stress. **a** Spearman’s rank correlation analyses between the measured parameters in *C. nodosa* leaves. **b** Example of the strongest correlations observed with the *F*_v_/*F*_m_ ratio. **c** Model explaining the possible role of vitamin E at the molecular level in photosynthetic membranes of *C. nodosa*

Some studies in *C. nodosa* suggest that its optimal growth temperature may oscillate between 24.5 °C (Peduzzi and Vukovic 1990; Lee et al. 2007) and 30–32 °C (Terrados and Ros 1995). Therefore, our study is in agreement with these previous results in relation to the temperatures at which *C. nodosa* suffers from photo-inhibition (both during autumn and winter, but particularly during the latter), and it further supports the contention that *C. nodosa* is very well adapted to warm temperatures. Low temperatures in plants can generate various types of stresses, including desiccation and osmotic stress, as well as oxidative stress, most particularly when combined with high solar radiation. Despite growing in waters with seasonal variations in light attenuation coefficients, plants received irradiances in excess of 1000 μmol m^−2^ s^−1^ of photosynthetically active photon flux density at noon in winter, spring and summer, well above the levels necessary to saturate photosynthesis, as previously shown in laboratory experiments (90–400 μmol m^−2^ s^−1^; Pérez and Romero 1992). In the present study, we showed that low temperatures induced desiccation in leaves, but not in rhizomes, and that both leaves and rhizomes were very tolerant to cold-induced oxidative stress. Although rhizomes experienced increased oxidative stress during winter, as indicated by enhanced LOOH contents during winter compared to autumn and spring, MDA contents did not increase, thus indicating absence of oxidative damage. Furthermore, it appeared that, despite the transient photoinhibition occurring in leaves during winter, an increased α-toc/Chl ratio led to an enhanced protection of the photosynthetic apparatus. The α-Toc/Chl ratio increased during winter mostly because of a reduction of Chl levels, thus indicating that Chl degradation at low temperatures may improve the antioxidant capacity of leaves per amount of photons absorbed, as it occurs in terrestrial plants (Kyparissis et al. 2000; Munné-Bosch and Alegre 2000; Fernández-Marín et al. 2017; Baccari et al. 2020). Interestingly, this is another ecological case study in which chlorophyll loss combined with enhanced antioxidant protection per amount of photons absorbed is associated with transient photoinhibition and full recovery from stress, in this case, low-temperature stress combined with relatively high solar radiation during winter in a seagrass, a model that was not investigated in this respect thus far. It is noteworthy that the *F*_v_/*F*_m_ ratio strongly negatively correlated with the α-toc/Chl ratio and the Chl *a*/*b* ratio, but not with markers of the extent of lipid peroxidation (Fig. 6a, b). Thus, adjustments in the pigment antenna size, consisting of a reduction of chlorophyll contents, but most particularly of Chl *b*, might help provide enhanced antioxidant protection, in terms of α-toc relative to the amount of photons absorbed in the antenna, thus leading to an overprotection from potential singlet oxygen damage in PSII (Fig. 6c, d). At this point, it is very difficult to discern whether the increase in the α-toc/Chl ratio led to reduced photo-inhibition, or if the occurrence of photo-inhibition caused an increased α-toc/Chl ratio, since it is known that singlet oxygen produces photo-inhibition, but also that α-toc could protect PSII from sustained photo-inhibitory damage (Kumar et al. 2021). One outlier showing *F*_v_/*F*_m_ values below 0.15 suggests that strong photo-inhibition may occur because of the inability of leaves to rapidly reduce Chl *b* levels that consequently lead to the absence of sufficiently increased α-toc/Chl ratio (Fig. 6b). However, further studies are necessary to study inter-clonal differences in photoinhibition, photo-oxidative stress and photoinhibitory damage in *C. nodosa* plants. It should be noted that these plants make new leaves constantly, most particularly during summer months, and they grow and colonize new space during each spring and summer making new clones (Pérez et al. 1994; Cancemi et al. 2002), which together with the fact that they adapt well to their environment, even to the cold temperatures experienced during winter, as shown in the present study, it is suggested that anthropogenic factors rather than environmental ones are the major risk these meadows are facing nowadays in a frame of global change.

In conclusion, our results indicate that (i) adjustments in pigment antenna size and vitamin E contents per unit of Chl during winter may help protect the photosynthetic apparatus from sustained photoinhibitory damage and lipid peroxidation events in leaves of *C. nodosa*, (ii) rhizomes also accumulate large amounts of vitamin E, although to a much lesser extent than leaves, and keep protected from lipid peroxidation during winter, as indicated by malondialdehyde contents, and (iii) vitamin E can help protect both leaves and rhizomes from lipid peroxidation in marine angiosperms, as it occurs in terrestrial plants, although cold stress during winter can cause transient photoinhibition of the photosynthetic apparatus in *C. nodosa*.

### *Author contribution statement*

SMB conceived of the project with the help of SP. SP performed samplings with the help of ERF. AC and ESF performed biochemical analyses. SMB and ESF analyzed and interpreted the results and wrote the manuscript. All authors revised and approved the manuscript.

## Data Availability

All of the raw data will be made available on reasonable request to the corresponding author.

## Abbreviations

α-toc: α-Tocopherol
Chl: Chlorophyll
*F*_v_/*F*_m_: Maximum efficiency of PSII photochemistry
LOOH: Lipid hydroperoxides
MDA: Malondialdehyde
ROS: Reactive oxygen species
